# Prognostic nomogram for small-cell lung cancer patients with brain metastasis in China: A population-based real-world analysis

**DOI:** 10.1097/MD.0000000000045367

**Published:** 2025-11-21

**Authors:** Weili Wang, Hongwei Li, Sufang Jia, Xiaqin Zhang, Wei Bai, Sijin Li

**Affiliations:** a Department of Radiotherapy, Shanxi Medical University, Shanxi Province Cancer Hospital, Cancer Hospital Affiliated to Shanxi Medical University, Shanxi Hospital Affiliated to Cancer Hospital, Chinese Academy of Medical Sciences, Taiyuan, China; b Department of Nuclear Medicine, Shanxi Medical University, The First Clinical Hospital Affiliated to Shanxi Medical University, Taiyuan, China.

**Keywords:** brain metastasis, case series, nomogram, overall survival, small-cell lung cancer

## Abstract

Small-cell lung cancer (SCLC) is one of the top malignancies that leads to brain metastasis (BM), and the prognosis for SCLC patients tends to be abysmal. However, there is a notable amount of heterogeneity among these patients, and a subset of them can be expected to survive much longer than average. Therefore, we aimed to develop a model to predict the prognosis of SCLC patients with BM. This retrospective study collected data from the medical records of 697 SCLC patients with brain malignancies treated at Shanxi Provincial Cancer Hospital from January 2008 to December 2018. Among them, 526 patients were used as the training cohort. Univariate and multivariate analyses were performed in this cohort to select independent risk factors that affect the prognosis of SCLC patients with BM, and a nomogram model was established. The remaining 171 patients were used as the validation cohort to verify the model. Our analysis showed that 7 pretreatment variables were related to the overall survival of SCLC patients with BM. These variables were incorporated into the model. Our predictive model performed well in validation and outperformed the lung diagnostic specific graded prognostic assessment. It may serve as a practical tool to guide individualized treatment decisions for SCLC patients with BM, helping to identify those suited for aggressive therapy versus supportive care. Future multicenter prospective studies are needed to validate its generalizability and explore optimized treatment strategies.

## 1. Introduction

Small-cell lung cancer (SCLC) is highly prone to brain metastasis (BM), which occurs in 15% to 20% of patients at initial diagnosis and in 40% to 50% during the disease course.^[1,2]^ The prognosis of these patients remains poor, with a median overall survival (OS) of only 5 to 11 months.^[3–5]^ Whole-brain radiotherapy (WBRT) is commonly used due to the frequent presence of multiple intracranial lesions,^[6]^ while palliative chemotherapy can modestly improve survival compared with supportive care alone.^[7]^ For patients with poor performance status, corticosteroids and best supportive care may be appropriate.^[8]^ As treatment decisions must balance survival benefits with risks such as neurocognitive impairment from WBRT,^[9]^ accurate prognostic tools are essential to guide clinical management.

Several prognostic indices have been developed, including recursive partitioning analysis,^[10]^ graded prognostic assessment (GPA), and the tumor–lymph node-metastasis (TNM) staging system.^[11]^ However, these tools do not distinguish between lung cancer subtypes, which differ in biology and clinical behavior. Nomograms, increasingly used in oncology, provide individualized predictions with higher accuracy than traditional staging systems.^[12–15]^ Barnholtz-Sloan et al proposed a BM nomogram for multiple primary cancers,^[16]^ but it lacked specificity for SCLC. More recently, Shan et al developed an SCLC-specific BM nomogram based on the SEER database,^[17]^ though its applicability to Chinese patients is uncertain.

The objective of this study was to identify key factors affecting OS in SCLC patients with BM and to develop a nomogram based on a large Chinese cohort. The innovations of this work are 3-fold. First, unlike previous models derived mainly from Western or SEER data, our study used real-world data from Chinese patients, ensuring greater local relevance. Second, the nomogram incorporated demographic, disease-related, laboratory (e.g., neutrophil-to-lymphocyte ratio [NLR]), and treatment factors, providing a comprehensive assessment. Third, we directly compared our model with the lung diagnostic specific (DS)-GPA system and demonstrated superior predictive accuracy, underscoring its value for individualized prognosis.

## 2. Methods

The present study was a retrospective analysis. The protocol of this study was approved by the Research Ethics Board of the Shanxi Cancer Research Institute (approval no. 20231018065;approval date: October 18, 2023) and was performed in compliance with the Declaration of Helsinki. As the study was retrospective and anonymous, the requirement for consent from each included patient was waived.

### 2.1. Patient selection

**Figure 1.**
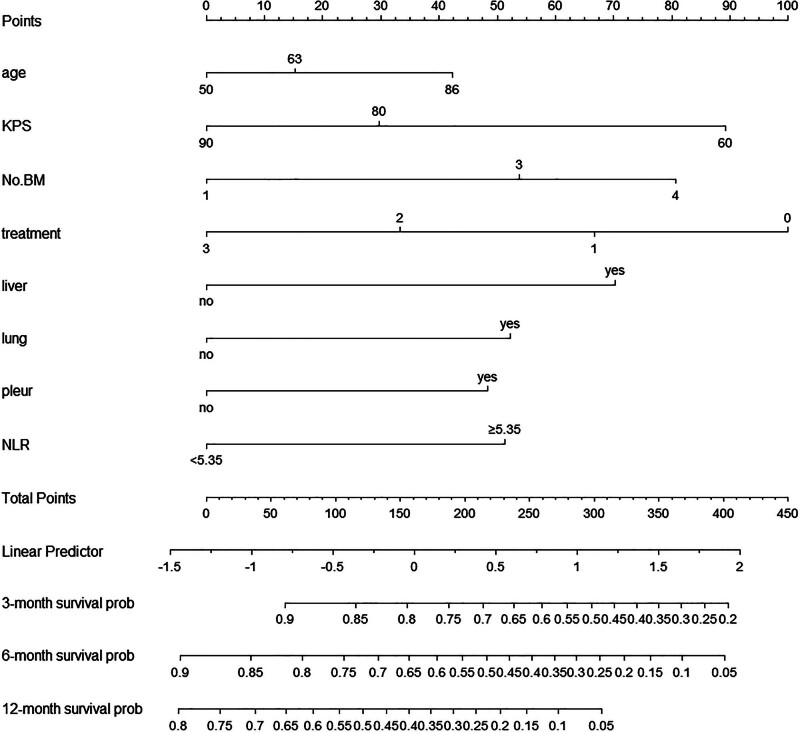
Nomogram predicting the 3-month, 6-month, and 12-month overall survival of patients with brain metastases from small-cell lung cancer.

We retrieved data from the digital medical records of Shanxi Provincial Cancer Hospital from January 2008 to December 2018. Patients with SCLC were screened. The inclusion criteria for patients were as follows: having a first and only primary cancer diagnosis of SCLC; having pathologically confirmed SCLC; and having BM found by contrast-enhanced computed tomography or gadolinium-enhanced magnetic resonance imaging at the initial diagnosis or during the course of treatment for this disease. The exclusion criteria included: having another malignant tumor(s); with incomplete clinical demographic variables and/or outcome data (e.g., age, sex, race, marriage status, TNM information, and survival time); and with less than a 3-month follow-up. We screened 3641 SCLC patients; finally, 697 eligible patients were included in the primary cohort. All eligible patients with BM from SCLC were categorized into the training group or validation group by the time of BM diagnosis. The patients diagnosed during the period of 2008 to 2015 (n = 526) were assigned to the training set, and those diagnosed from 2016 to 2018 (n = 171) were assigned to the validation set (Fig. 1). The training group was used to develop the nomogram, while the validation group was used to externally validate its applicability.

### 2.2. Study variables

The following data of the included patients were retrieved from the hospital medical records: demographics (i.e., age at diagnosis, sex, smoking status, and year at diagnosis), clinical characteristics (i.e., Karnofsky index of performance status [KPS], extracranial metastasis (ECM), number of BMs, sites of ECM [e.g., liver, bone, adrenal glands, or others], and NLR), as well as treatment information, which was categorized into 4 modalities: best supportive care, WBRT-only, chemotherapy-only, and WBRT plus chemotherapy.

The primary endpoint was OS, which was defined as the time from the day of diagnosis of BM to the day of death or loss of follow-up. The best cutoff values for age and NLR were defined by X-tile software.

### 2.3. Statistical methods

The frequency distributions of demographic information, clinical characteristics, and treatment information of eligible patients were calculated separately for the training set and the validation set. The nomogram was created based on independent prognostic factors using the “rms” function in the R package. Cox proportional hazards regression models (both univariate and multivariate) were used to calculate hazard ratios with 95% confidence intervals for independent prognostic factors of OS using the training cohort. The target variables in the univariate COX regression model included the age at BM diagnosis (20–50 vs 51–63 vs 64–86 years old; the best cutoff value for age was defined by X-tile software), sex (female vs male), smoking status (yes vs no), KPS (<70 vs 70–80 vs 90–100), ECM (yes vs no), number of BM lesions (1 vs 2/3 vs >3), treatment regimen (best supportive care vs WBRT-only vs chemo-only vs WBRT + chemo), site of ECM (e.g., liver, bone, lung, renicapsule, and pleura), and NLR (0.02–5.33 vs 5.35–63.86; the best cutoff value for age was defined by X-tile software). For variables with missing values, cases with incomplete key clinical or survival information were excluded. For variables with a small proportion of missing values (<5%), missing entries were imputed using the median value to minimize data loss.

We set *P*-value < .05 as statistical significance. Significant variables in the univariate Cox regression analyses were included in the multivariate Cox regression analysis. The significant variables found in the multivariate model were used to establish the nomogram and risk classification system.

The 3-month, 6-month, and 12-month OS rates were the primary endpoints of this study. The calibration curves employed with bootstrapping to decrease the bias of over-fitting and the area under the time-dependant receiver operating characteristic curve were used to estimate the discrimination of the predictive model both in the training and validation settings.^[18]^

Furthermore, the clinical performance of the nomogram was compared with the previously published prognostic model lung DS-GPA, as seen in Table 1. The C-index values of the present nomogram model and the lung DS-GPA were calculated. Meanwhile, decisive curve analyses of the 3-month, 6-month, and 12-month OS rates were applied to estimate the clinical values between the 2 prognostic systems. All statistical tests were performed using R software (version 3.5.1; Posit, PBC [formerly RStudio, PBC], Boston).

**Table 1:** Performance of the 2 models.

	C-index	C-*P*
Train		<.001
Model 1	0.702	
GPA group	0.625	
Test		
Model 1	0.688	
GPA group	0.561	

Model 1: The multifactor nomogram model.

C-index = consistency index, C-*P* = *P*-value of the consistency index, GPA = graded prognostic assessment, NLR = neutrophil-to-lymphocyte ratio.

### 2.4. Reporting

This case series was reported in accordance with the PROCESS guidelines.^[19]^

## 3. Results

### 3.1. Participants

According to our inclusion and exclusion criteria, we finally included 697 eligible patients with BM from SCLC in the final analysis. Then, 526 and 171 patients were grouped into the training and validation sets, respectively. The median follow-up time was 8 months (range: 1–16 months).

The clinicopathological characteristics and baseline data are provided in Table 2. The treatment regimens were categorized into 4 groups: best supportive care (BSM), WBRT-only, chemotherapy-only, and WBRT plus chemotherapy concurrently or sequentially (Table 2).

**Table 2:** The characteristics of the included patients in the final analysis.

	Training cohort		Validation cohort	
Variable	N = 526		N = 171	
	n	%	n	%
Sex				
Male	430	81.7	143	83.6
Female	96	18.3	28	16.4
Age (yr)				
20–50	110	20.9	26	9.6
51–63	171	32.5	98	57.3
64–86	245	46.6	47	27.5
Smoker				
No	137	26.0	37	21.6
Yes	389	74.0	134	78.4
KPS				
<70	66	12.5	27	15.8
70–80	274	52.1	68	39.8
90–100	186	35.4	76	44.4
ECM				
No	322	61.2	105	61.4
Yes	204	38.8	66	38.6
No. of BMs				
1	193	36.7	55	32.2
2–3	126	24.0	44	25.7
>3	207	39.4	72	42.1
Primary status				
No	223	42.4	64	37.4
Yes	303	57.6	107	62.6
Treatment				
BSC	98	18.6	20	11.7
WBRT-only	141	26.8	37	21.6
Chemo-only	78	14.8	51	29.8
WBRT + Chemo	209	39.7	63	36.8
Liver metastasis				
No	458	87.1	139	81.3
Yes	68	12.9	32	18.7
Renicapsular metastasis				
No	462	87.8	128	74.9
Yes	64	12.2	43	25.1
Bone metastasis				
No	428	81.4	141	82.5
Yes	98	18.6	30	17.5
Lung metastasis				
No	492	93.5	154	90.1
Yes	34	6.5	16	9.4
Pleural metastasis				
No	452	85.9	146	85.4
Yes	74	14.1	25	14.6
NLR				
0.02–5.33	443	84.2	147	86.0
5.35–63.86	83	15.8	24	14.0

BM = brain metastasis, BSC = best supportive care, ECM = extracranial metastasis, KPS = Karnofsky performance status score, NLR = neutrophil-to-lymphocyte ratio, No. of BMs = number of brain metastases, WBRT = whole-brain radiotherapy.

### 3.2. Development and validation of the prognostic nomogram

According to Cox regression analysis, the independent predictors for OS in this setting included age, KPS, number of BMs, NLR, liver metastasis, lung metastasis, pleural metastasis, and treatment modality (Table 3). The prognostic nomogram for the 3-month, 6-month, and 12-month OS is presented in Figure 1. Each independent factor was allocated a score on the point scale. The sum of the scores was used to estimate the 1-year, 2-year, and 3-year OS of individual patients.

**Table 3:** Univariate and multivariate analysis.

Variable	Univariate analysis		Multivariate analysis	
	HR (95% CI)	*P*	HR (95% CI)	*P*
Sex (male vs female)	1.228 (0.970–1.554)	.088	–	–
Age (20–50 vs 51–63 vs 64–86 years old)	1.010 (1.003–1.017)	.003	1.010 (1.003–1.017)	.006
Smoker (no vs yes)	1.184 (0.962–1.458)	.111	–	–
KPS (<70 vs70–80 vs 90–100)	0.971 (0.961–0.981)	<.001	0.978 (0.968–0.988)	<.001
ECM (no vs yes)	0.652 (0.540–0.788)	<.001	–	–
No. of BMs (1 vs 2–3 vs > 3)	1.225 (1.142–1.314)	<.001	1.233 (1.147–1.325)	<.001
Primary status (no vs yes)	0.807 (0.672–0.970)	.022	–	–
Treatment (BSC vs WBRT-only vs Chemo-only vs WBRT + Chemo)	0.783 (0.725–0.846)	<.001	0.768 (0.708–0.833)	<.001
Liver (no vs yes)	2.086 (1.603–2.713)	<.001	1.582 (1.184–2.113)	.002
Renicapsular metastasis (no vs yes)	1.225 (0.922–1.627)	.162	–	–
Bone metastasis (no vs yes)	1.542 (1.225–1.940)	<.001	–	–
Lung metastasis (no vs yes)	1.648 (1.149–2.362)	.007	1.503 (1.036–2.180)	.032
Pleural metastasis (no vs yes)	1.472 (1.137–1.907)	.003	1.333 (1.007–1.764)	.045
NLR (0.02–5.33 vs 5.35–63.86)	1.749 (1.374–2.227)	<.001	1.433 (1.113–1.845)	.005

BM = brain metastasis, BSC = best supportive care, CI = confidence interval, ECM = extracranial metastasis, HR = hazard ratio, KPS = Karnofsky Performance Status score, NLR = neutrophil-to-lymphocyte ratio, No. of BMs = number of brain metastases, WBRT = whole-brain radiotherapy.

**Figure 2.**
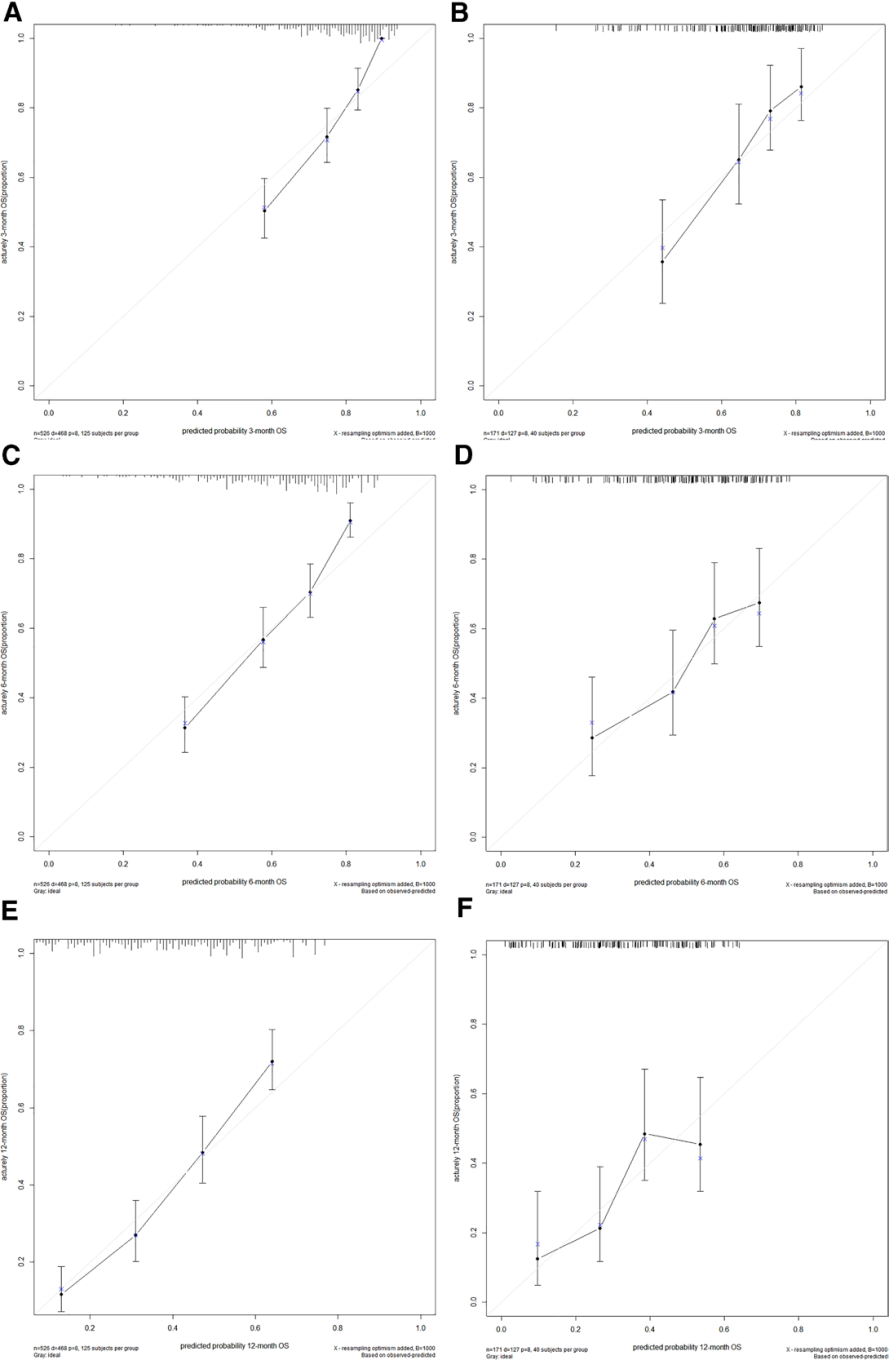
Calibration curves. The calibration curves of the nomogram for predicting the 3-month (A, D), 6-month (B, E), and 12-month (C, F) overall survival of the training set and of the validation set, respectively.

**Figure 3.**
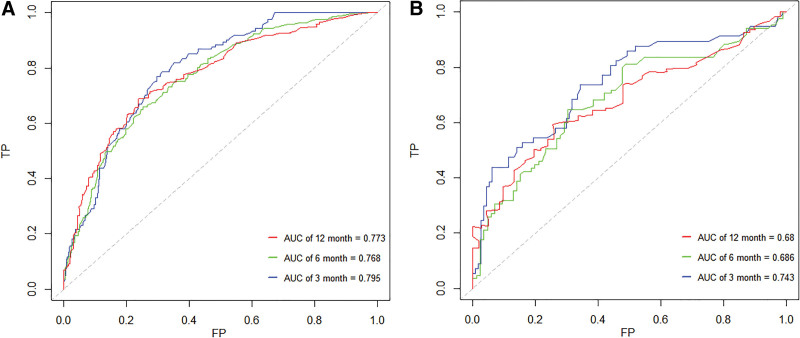
Receiver operating characteristic (ROC) curves of the nomogram for predicting the overall survival of patients with brain metastases from small-cell lung cancer. ROC curves for the 1-year, 2-year, and 3-year overall survival in the training set (A); ROC curves for predicting the 3-month, 6-month, and 12-month overall survival in the validation set (B).

The nomogram was validated in the training group and validation group, respectively. The calibration plots for the probability of OS showed a good agreement at the 3-month, 6-month, and 12-month time points between the predicted and actual results in both the training and validation groups (Fig. 2). The area under curve values of the receiver operating characteristic curves for the training cohort for the 3-month, 6-month, and 12-month OS were 0.795, 0.768, and 0.773, respectively. While for the validation cohort, they were 0.743, 0.686, and 0.68, respectively (Fig. 3).

### 3.3. Comparison of the prognostic nomogram for SCLC patients with BM with the lung DS-GPA

**Figure 4.**
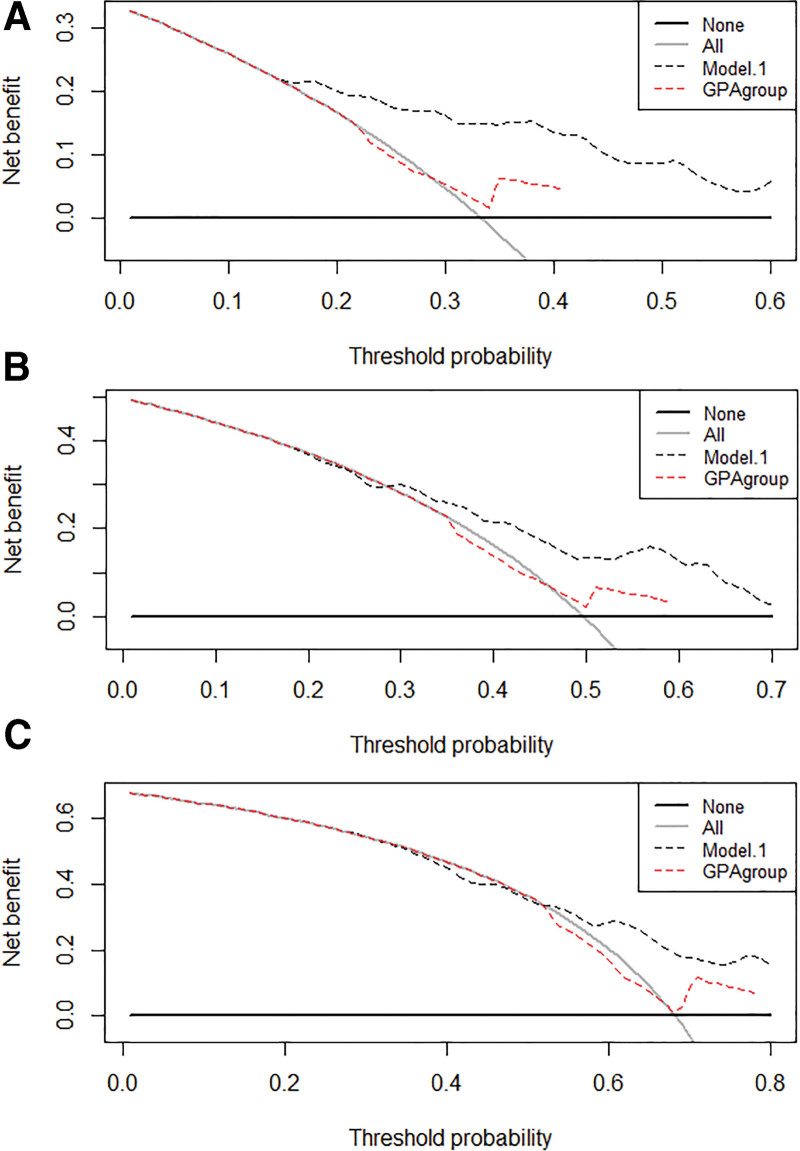
Decision curve analysis for predicting the 3-month (A), 6-month (B), and 12-month (C) overall survival in the validation set using our model (model 1) versus DS-GPA. Model 1 included all the factors in the nomogram, and DS-GPA was the diagnosis-specific graded prognostic assessment (GPA group). DS = diagnostic specific, GPA = graded prognostic assessment.

According to decisive curve analyses, it was determined that the prognostic nomogram we generated showed a greater net benefit than the lung DS-GPA system for predicting the 3-month, 6-month, and 12-month OS when the threshold probability was more than 0, 15, 0.25, and 0.26, respectively (Fig. 4).

## 4. Discussion

SCLC is a type of lung cancer that is especially prone to metastasis, with approximately 70% of patients having distant metastasis at their primary diagnosis.^[20]^ Among the metastatic sites, the brain is particularly vulnerable, with more than 10% of patients presenting with BM at their first visit.^[21]^ Patients with metastatic SCLC typically survive for about a year, while those with BM survive for only around 5 months.^[22]^ Although several prognostic tools such as GPA, recursive partitioning analysis, and TNM staging exist, none are specifically tailored to SCLC patients with BM. Using a relatively large cohort, our study developed a nomogram that addresses this clinical gap.

Several variables were identified as significant prognostic factors. KPS reflects patients’ functional reserve and treatment tolerance, which directly influence survival outcomes.^[23]^ NLR, as a systemic inflammation marker, may promote tumor progression through immune suppression and tumor microenvironment modulation, explaining its association with poor prognosis.^[24]^ Smoking status, a reflection of both lifestyle and underlying tumor biology, was also predictive of inferior survival. Moreover, extracranial metastases—particularly in the liver, lung, and pleura—likely indicate higher tumor burden and more aggressive disease biology, resulting in worse OS. These findings highlight the multifactorial nature of prognosis in SCLC with BM and support the inclusion of such variables in individualized prediction tools.

Beyond identifying prognostic variables, our study provides practical implications for treatment decision-making. The nomogram can help clinicians distinguish patients who may benefit from aggressive therapies such as WBRT plus chemotherapy from those more suitable for supportive or palliative car. For example, patients with favorable prognostic features (e.g., higher KPS, lower NLR, and absence of extracranial metastases) may derive meaningful survival benefit from combined-modality treatment, while those with poor prognostic profiles may avoid unnecessary treatment-related neurocognitive toxicity and instead focus on symptom relief and quality of life. Thus, the model not only predicts survival but also serves as a reference for tailoring treatment strategies. The DCA further highlighted the clinical utility of our model by showing a superior net benefit across a range of clinically relevant threshold probabilities. This implies that the nomogram may aid physicians in balancing the risks and benefits of aggressive treatment strategies versus supportive care in patients with SCLC and BM.

Our study has several strengths. A relatively large sample was used to construct and validate the predictive model, which demonstrated good calibration and discrimination compared with the DS-GPA. Nevertheless, several limitations should be acknowledged. First, its retrospective nature introduces the potential for selection bias. Second, the long inclusion period (2008–2018) may have introduced time-related bias, as diagnostic protocols, treatment strategies, and supportive care have evolved over this decade. Although we attempted to mitigate this by dividing patients into training and validation cohorts, residual confounding from temporal changes cannot be excluded. Third, differences in treatment regimens and supportive measures during this period—such as advances in chemotherapy, improvements in radiotherapy, and better symptom management—were not fully stratified in our analysis and may have influenced outcomes. Finally, as the model was internally validated in a single center and specific to SCLC with BM, its generalizability remains limited. Future studies involving external, multi-center, and prospective validation with more detailed treatment information are needed to confirm the robustness and broader applicability of our model.

In summary, the nomogram developed in this study provides a more accurate and clinically relevant tool for prognostication in SCLC patients with BM. It allows for the identification of patients with relatively favorable survival prospects and may guide personalized treatment planning, particularly in balancing aggressive therapy with supportive care. Future prospective studies are needed to validate the model and to explore its utility in optimizing radiotherapy and chemotherapy strategies.

## 5. Conclusions

In this study, we developed and validated a prognostic nomogram for SCLC patients with BM using a large real-world cohort. The model demonstrated good calibration and discrimination and outperformed the lung DS-GPA in predicting short-term survival. Beyond providing individualized survival estimates, this tool may assist clinicians in identifying patients who could benefit from more aggressive multimodality therapy versus those better suited for supportive care. Nevertheless, further external and prospective validation is warranted to confirm its generalizability and to refine its role in guiding clinical decision-making.

## Acknowledgments

We thank Medjaden Inc. for scientific editing of this manuscript.

## Author contributions

**Conceptualization:** Weili Wang, Wei Bai, Sijin Li.

**Data curation:** Wei Bai, Sijin Li.

**Formal analysis:** Hongwei Li, Sijin Li.

**Investigation:** Hongwei Li, Sijin Li.

**Methodology:** Sufang Jia, Sijin Li.

**Validation:** Sufang Jia, Sijin Li.

**Visualization:** Xiaqin Zhang, Sijin Li.

**Writing – original draft:** Weili Wang, Sijin Li.

**Writing – review & editing:** Weili Wang, Sijin Li.

## References

[1] Zhu Y Cui Y Zheng X Zhao Y Sun G. Small-cell lung cancer brain metastasis: from molecular mechanisms to diagnosis and treatment. Biochim Biophys Acta Mol Basis Dis. 2022;1868:166557. 36162624 10.1016/j.bbadis.2022.166557

[2] van Meerbeeck JP Fennell DA De Ruysscher DK. Small-cell lung cancer. Lancet. 2011;378:1741–55. 21565397 10.1016/S0140-6736(11)60165-7

[3] Amgen Inc. Tarlatamab-dlle (Imdelltra) significantly reduces risk of death by 40% versus chemotherapy in previously treated extensive-stage small cell lung cancer: phase III trial results. J Clin Oncol. 2025;43:13.

[4] Bernhardt D Adeberg S Bozorgmehr F. Outcome and prognostic factors in patients with brain metastases from small-cell lung cancer treated with whole brain radiotherapy. J Neurooncol. 2017;134:205–12. 28560661 10.1007/s11060-017-2510-0

[5] Bernhardt D Adeberg S Bozorgmehr F. Outcome and prognostic factors in single brain metastases from small-cell lung cancer. Strahlenther Onkol. 2018;194:98–106. 29085978 10.1007/s00066-017-1228-4

[6] Xiao S Mei Z Xie Z Lu H. Development and validation of nomograms for predicting survival in small cell lung cancer patients with brain metastases: a SEER population-based analysis. Am J Transl Res. 2024;16:2318–33. 39006302 10.62347/TLWB3988 PMC11236647

[7] Rong YT Zhu YC Wu YA. A novel nomogram predicting cancer-specific survival in small cell lung cancer patients with brain metastasis. Transl Cancer Res. 2022;11:4289–302. 36644187 10.21037/tcr-22-1561 PMC9834596

[8] Solomon BJ Liu G Felip E. Lorlatinib versus crizotinib in patients with advanced ALK-positive non-small cell lung cancer: 5-year outcomes from the phase III CROWN study. J Clin Oncol. 2024;42:3400–9. 38819031 10.1200/JCO.24.00581 PMC11458101

[9] Vogelbaum MA Brown PD Messersmith H. Treatment for brain metastases: ASCO-SNO-ASTRO guideline. J Clin Oncol. 2022;40:492–516. 34932393 10.1200/JCO.21.02314

[10] Curran WJ Jr Scott CB Horton J. Recursive partitioning analysis of prognostic factors in three radiation therapy oncology group malignant glioma trials. J Natl Cancer Inst. 1993;85:704–10. 8478956 10.1093/jnci/85.9.704

[11] Zindler JD Rodrigues G Haasbeek CJ. The clinical utility of prognostic scoring systems in patients with brain metastases treated with radiosurgery. Radiother Oncol. 2013;106:370–4. 23522151 10.1016/j.radonc.2013.01.015

[12] Han D-S Suh Y-S Kong S-H. Nomogram predicting long-term survival after D2 gastrectomy for gastric cancer. J Clin Oncol. 2012;30:3834–40. 23008291 10.1200/JCO.2012.41.8343

[13] Zaak D Burger M Otto W. Predicting individual outcomes after radical cystectomy: an external validation of current nomograms. BJU Int. 2010;106:342–8. 20002664 10.1111/j.1464-410X.2009.09138.x

[14] Karakiewicz PI Briganti A Chun FKH. Multi-institutional validation of a new renal cancer–specific survival nomogram. J Clin Oncol. 2007;25:1316–22. 17416852 10.1200/JCO.2006.06.1218

[15] Su D Zhou X Chen Q. Prognostic nomogram for thoracic esophageal squamous cell carcinoma after radical esophagectomy. PLoS One. 2015;10:e0124437. 25893524 10.1371/journal.pone.0124437 PMC4404051

[16] Barnholtz-Sloan JS Yu C Sloan AE. A nomogram for individualized estimation of survival among patients with brain metastasis. Neuro Oncol. 2012;14:910–8. 22544733 10.1093/neuonc/nos087 PMC3379797

[17] Shan Q Shi J Wang X. A new nomogram and risk classification system for predicting survival in small cell lung cancer patients diagnosed with brain metastasis: a large population-based study. BMC Cancer. 2021;21:640. 34051733 10.1186/s12885-021-08384-5 PMC8164795

[18] Alba AC Agoritsas T Walsh M. Discrimination and calibration of clinical prediction models: users’ guides to the medical literature. JAMA. 2017;318:1377–84. 29049590 10.1001/jama.2017.12126

[19] Agha RA Sohrabi C Mathew G Franchi T Kerwan A O'Neill N. The PROCESS 2020 guideline: updating consensus preferred reporting of CasESeries in surgery (PROCESS) guidelines. Int J Surg. 2020;84:231–5. 33189880 10.1016/j.ijsu.2020.11.005

[20] Rudin CM Brambilla E Faivre-Finn C Sage J. Small-cell lung cancer. Nat Rev Dis Primers. 2021;7:3. 33446664 10.1038/s41572-020-00235-0 PMC8177722

[21] Saeed NA Jin L Sasse AW. Hypofractionated vs. standard radiotherapy for locally advanced limited-stage small cell lung cancer. J Thorac Dis. 2022;14:306–20. 35280466 10.21037/jtd-21-1566 PMC8902118

[22] Siegel RL Miller KD Jemal A. Cancer statistics, 2018. CA Cancer J Clin. 2018;68:7–30. 29313949 10.3322/caac.21442

[23] Firat S Bousamra M Gore E Byhardt RW. Comorbidity and KPS are independent prognostic factors in stage I non-small-cell lung cancer. Int J Radiat Oncol Biol Phys. 2002;52:1047–57. 11958901 10.1016/s0360-3016(01)02741-9

[24] Valero C Lee M Hoen D. Pretreatment neutrophil-to-lymphocyte ratio and mutational burden as biomarkers of tumor response to immune checkpoint inhibitors. Nat Commun. 2021;12:729. 33526794 10.1038/s41467-021-20935-9 PMC7851155

